# Safety and Efficacy of Peptide-Receptor Radionuclide Therapy in Elderly Neuroendocrine Tumor Patients

**DOI:** 10.3390/cancers13246290

**Published:** 2021-12-15

**Authors:** Deborah Theiler, Marco Cattaneo, Lawrence O. Dierickx, Peter Igaz, Simona Grozinsky-Glasberg, Claire Bournaud, Thomas O’Dorisio, M. Sue O’Dorisio, Damian Wild, Emanuel Christ, Guillaume P. Nicolas

**Affiliations:** 1 Division of Nuclear Medicine, University Hospital of Basel, 4031 Basel, Switzerland; deborah.theiler@unibas.ch (D.T.); damian.wild@usb.ch (D.W.); 2 Department of Clinical Research, University of Basel, 4031 Basel, Switzerland; Marco.Cattaneo@usb.ch; 3 Department of Nuclear Medicine, ENETS CoE, Institut Universitaire du Cancer Toulouse-Oncopole, 31100 Toulouse, France; Dierickx.Lawrence@iuct-oncopole.fr; 4 Department of Endocrinology, Department of Internal Medicine and Oncology, ENETS CoE, Semmelweis University, 1085 Budapest, Hungary; igaz.peter@med.semmelweis-univ.hu; 5 MTA-SE Molecular Medicine Research Group, Hungarian Academy of Sciences and Semmelweis University, 1085 Budapest, Hungary; 6 Neuroendocrine Tumor Unit, Department of Endocrinology and Metabolism, ENETS CoE, Hadassah-Hebrew University Medical Center, Jerusalem 9112001, Israel; simonag@hadassah.org.il; 7 Department of Nuclear Medicine, Hospices Civils de Lyon, 69310 Lyon, France; claire.bournaud@ch-roanne.fr; 8 Department of Endorinology, Stead Family Children’s Hospital, University of Iowa, Iowa City, IA 52242, USA; thomas-odorisio@uiowa.edu (T.O.); sue-odorisio@uiowa.edu (M.S.O.); 9 Neuroendocrine and Endocrine Tumour Centre, ENETS CoE, University Hospital of Basel, 4031 Basel, Switzerland; Emanuel.christ@usb.ch; 10 Department of Endocrinology, University Hospital of Basel, 4031 Basel, Switzerland

**Keywords:** neuroendocrine tumour, peptide receptor radionuclide therapy, elderly patients, ^177^Lu-DOTATOC, ^90^Y-DOTATOC

## Abstract

**Simple Summary:**

We compared the safety and efficacy of targeted radionuclide therapy between elderly (79 years old and older) and disease-matched younger patients (between 60 and 70 years of age) with metastatic neuroendocrine tumour (NET). To our knowledge, this is the first paper addressing this important clinical question of the outcome of radionuclide therapy in this particularly vulnerable population. We found that targeted radionuclide therapy did not cause increased side effects in the elderly NET population, while toxicity remains modest and comparable in both groups. We also find that survival (after adjusting for differences in life expectancy) is not inferior for the elderly compared to younger NET patients.

**Abstract:**

Peptide receptor radionuclide therapy (PRRT) is a well-established treatment in somatostatin receptor-expressing neuroendocrine tumours (NETs). The safety and efficacy of PRRT in >79 years old patients (EP) have not been systematically investigated. All patients with inoperable/metastatic/progressive G1/G2 NET, >79 years (EP), treated with PRRT at the University Hospital of Basel between 2006 and 2018, were enrolled in this retrospective matched cohort study. Each patient was manually matched with ≥1 younger patient (YP = 60–70 years). The primary endpoint was toxicity. Toxicity (subacute, long-term) was graded according to the criteria for adverse events (CTCAE) v5.0. All toxicity grades ≥ 3, or whose delta (Δ) to baseline were ≥2, were considered significant. The odds ratio (OR) for developing toxicity was tested for non-inferiority of EP vs. YP. Clinical response to PRRT and overall survival (OS) were assessed as secondary outcome measures. Forty-eight EP and 68 YP were enrolled. Both cohorts were balanced regarding median time since diagnosis, tumour location, grading, treatment scheme, and baseline biochemical parameters, except for eGFR (EP: 61 ± 16 vs. YP: 78 ± 19; mL/min/1.73 m^2^). Twenty-two grade ≥ 3 or Δ ≥ 2 subacute hematotoxicities occurred in 10 EP (10.3% of cycles) and 37 in 19 YP (11.6% of cycles; *p* = NS). Long-term grade ≥ 3 renal toxicity occurred in 7 EP and 2 YP (*p* = NS). The median OS was 3.4 years (EP) vs. 6.0 years (YP), HR: 1.50 [0.75, 2.98], *p* = NS. PRRT is a valid therapeutic option in elderly NET patients with similar toxicity and non-inferior survival compared to matched younger patients.

## 1. Introduction

Neuroendocrine tumours (NETs) frequently overexpress somatostatin receptors, in particular subtype 2 (sst2), on the tumour cell membrane. These somatostatin receptors represent important molecular targets for imaging and therapy, using either radiolabelled or “cold” peptide analogues [1,2,3,4,5,6,7]. Peptide receptor radionuclide therapy (PRRT) with ^90^yttrium- or ^177^lutetium-labelled somatostatin analogues such as DOTATOC or DOTATATE has proven its safety and efficacy in patients with inoperable/metastatic gastroenteropancreatic NETs [2,4,5,7,8]. In particular, the recently published phase III trial (NETTER-1) documented the superiority of PRRT over “cold” somatostatin analogues in midgut NET in terms of objective response, progression-free survival and in improving quality of life.

In the NETTER-1 trial, the mean age was only 63 ± 9 years and 83% of the patients tolerated 3 or 4 fractions of PRRT with ^177^Lu-DOTATATE well with moderate, mostly reversible haematological and without significant renal toxicity [2,9]. However, elderly patients (EP) more frequently have comorbidities, exhibit an impaired glomerular filtration rate, and have a reduced haematological reserve and may, therefore, be at increased risk for PRRT-induced toxicity This may explain why EP are less frequently treated with PRRT [10]. On the other hand, the alternative systemic therapeutical approach with targeted therapies in metastatic NETs have significantly more side effects [4,11], which may impair quality of life [4,11]. Quality of life is of particular importance in EP. Thus, in EP an efficient well-tolerated systemic therapy is warranted. However, the safety and efficacy of PRRT in EP has not been systematically investigated. We, therefore, aimed at investigating the safety and efficacy of personalised and fractionated PRRT with yttrium-90 or lutetium-177-DOTATOC in NET patients >79 years in a retrospective matched cohort study.

## 2. Materials and Methods

### 2.1. Patients

All adult patients who presented locally inoperable, metastatic and progressive, histologically confirmed well-differentiated low (G1) or intermediate grade (G2) NETs with positive sst2 imaging were enrolled. Eligible patients were included if they underwent at least one cycle of PRRT at the University Hospital of Basel between 2006 and 2018, were aged >79 years for the elderly cohort and between 60 and 70 years for the younger cohort, respectively. The age range of the younger patients’ cohort (YP) corresponds to the typical age at which NET patients are diagnosed and treated [2,12,13]. Primary tumours were located in the pancreas, midgut (defined as the small intestine, appendix, ascending colon and proximal two-thirds of the transverse colon), hindgut (defined as the distal third of the transverse colon, descending colon, sigmoid colon and rectum), lungs or were of unknown origin. Well-differentiated histologic features were defined according to the 2019 WHO classification of tumours of the digestive system [14].

PRRT was indicated according to the applicable guidelines at the time the study was conducted [15,16].

Inclusion criteria: locally inoperable, metastatic and progressive, histologically confirmed well-differentiated low (G1) or intermediate grade (G2) NETs with positive somatostatin receptor imaging

Exclusion criteria: Patients with poorly differentiated neuroendocrine carcinoma, ECOG performance status ≥ 3 or enable to be transported, insufficient tumour uptake as visualised on somatostatin receptor imaging or inadequate laboratory parameters at baseline (mainly haemoglobin < 80 g/L, platelet count < 75 G/L, ASAT/ALAT > 3 times upper range of normal or eGFR < 45 mL/min) were contra-indicated to receive any PRRT. Patients were excluded if they had a non-NET-histology, histology of grade 3 NET or grade 3 neuroendocrine carcinoma. PRRT was also contraindicated in case of ECOG performance status ≥ 3 or if patients were not transportable, if there was insufficient tumour uptake on somatostatin receptor imaging or inadequate laboratory parameters at baseline (mainly haemoglobin < 80 g/L, platelet count < 75 G/L, ASAT/ALAT > 3 times upper range of normal or eGFR < 45 mL/min).

Patients with symptoms of carcinoid syndrome (diarrhoea or flush) and increased plasma or urinary-5-hydroxyindole acetic acid as well as symptoms of secreting pancreatic NET (gastrinoma, insulinoma etc.) were classified as having a functional tumour.

### 2.2. Trial Design

The patients of the elderly cohort were manually matched with at least one patient of the younger cohort, as matching with a variable number of controls generally increases the statistical precision [17]. Elderly Index cases were matched with younger controls based on the following criteria: sex, location of the primary tumour, grading, hormonal secretion (separated in functional and non-functional) and tumour load which was separated in small-volume disease, defined as pure nodal, or limited liver metastases (i.e., ≤25% of liver tissue involvement and including small peritoneal deposits), and wide-spread metastases with bulky or multiorgan involvement including mainly liver and bone metastasis.

### 2.3. Treatment Protocol

The therapy consisted of standardised treatment schemes of 2 to 4 PRRT cycles at 10 to 12-week intervals using 7.4 GBq ^177^Lu-DOTATOC and/or 3.7 GBq/m^2 90^Y-DOTATOC per cycle, depending on individual tumour burden estimate and laboratory test results, as previously published [7,18]; briefly: at least 1 cycle of ^90^Y-DOTATOC if estimated glomerular filtration rate (eGFR) was >60 mL/min and tumours were larger than 2 cm in diameter, 2 cycles if only ^90^Y-DOTATOC was administered, 3 cycles in total when ^90^Y-DOTATOC was used in combination with 2 cycles of ^177^Lu-DOTATOC, and 3 to 4 cycles if only ^177^Lu-DOTATOC was used. Besides ^90^Y-DOTATOC, where activity is already individually adapted depending on body habitus and administered only in case of (sub-)normal haematological and renal function, possible dose reduction was performed for ^177^Lu-DOTATOC depending on the patient’s clinical status and laboratory parameters. After cycle 1, a 25% dose reduction was implemented in case of not fully recovered previous G2 toxicity. Patients were disqualified from receiving subsequent treatment cycles in case of grade 3 or more haematological toxicity (except for lymphocytopenia) or eGFR < 45 mL/min. All patients received, as a reno-protective solution, 1 L of 2.5% arginine and 2.5% lysine infusion over 4 h starting 30–60 min prior to the radiopharmaceutical injection. Somatostatin analogue was continued in all patients with functional syndromes between and after PRRT.

### 2.4. Endpoints and Assessments

The primary endpoint was the safety and tolerability of PRRT with a special focus on kidney and bone marrow function. The differential blood cell count (haemoglobin, thrombocytes, leucocytes, neutrophil granulocytes and lymphocytes) and eGFR were measured at baseline, every second week after each PRRT cycle and 1, 2 and 3 years after completion of PRRT. Toxicities were defined as either subacute or long-term toxicity, if adverse events occurred within 12 weeks after a PRRT cycle or at least 1 year after the start of PRRT, respectively. Subacute and long-term toxicity were graded according to the U.S. National Cancer Institute Common Terminology Criteria for Adverse Events (CTCAE) v5.0. All toxicities grade ≥ 3, or whose delta (Δ: increase in toxicity grade) to baseline was ≥2 for hematotoxicities, were considered significant. Odds ratio (OR) or the occurrence of toxicity between cohorts were tested for non-inferiority. A decreased lymphocyte count is frequently encountered after PRRT and is extremely rarely clinically relevant. Lymphocyte toxicity in PRRT is mainly due to the selective targeting of B-cells and the relative sparing of T-lymphocytes could explain the absence of clinical side effects in these patients, such as the increased risk of infections. Due to this fact lymphocyte toxicity was not counted as part of hematotoxicities [19].

Efficacy of PRRT was assessed with overall survival (OS) and clinical response to PRRT as secondary outcome measures. The OS was calculated from the start of the first PRRT intervention to the last contact (right censoring) or the date of death from any cause, respectively. To correct for differences in the residual life expectancy of the two study cohorts, the standard time-scale was transformed to a reference-relative time-scale (% of the residual life-expectancy) based on the statistically expected life-span of the patient in relation to the Swiss population (depending on sex, year of birth and age at the start of treatment date).

The clinical response to PRRT was assessed after the completion of the therapy by asking family physicians or NET specialists about their overall opinion about the patient’s clinical benefit from PRRT based on all available clinical, biochemical and imaging data, scaled in 4 categories: no benefit, minor benefit, moderate benefit or major benefit, see Figure S1.

### 2.5. Statistical Analysis

All analyses were conducted using the statistical software package R, using two-sided confidence intervals with a standard confidence level of 95%.

Due to the retrospective nature of the study, there is no per-protocol set that differs from the full analysis set. Hence, all statistical analyses were performed on the full analysis set, although the main goal was to show non-inferiority with regard to safety and OS.

As some values of the primary endpoint were missing, all primary analyses were done once without these values (complete-case analyses) and repeated after imputing 100 times missing values by chained equations based on the missing at random (MAR) assumption [20]. The imputation of the primary endpoint was based on age, age group, sex, primary NET location, NET grading and the baseline values of the following laboratory parameters: haemoglobin, thrombocytes, leucocytes, neutrophil granulocytes, lymphocytes and eGFR. The following regression analyses were performed, always with the age group as the explanatory variable and with random effects for the matching strata: For each of the 6 laboratory parameters cited previously, 2 logistic regressions for the corresponding subacute adverse events (1 for CTCAE ≥ 3 and 1 for CTCAE Δ ≥ 2) with the patient as a nested random effect, and analogously 2 logistic regressions for the long-term adverse events. In all 22 logistic regressions, the non-inferiority of older vs. younger patients was tested by comparing the 95% confidence interval for the odds ratio (OR) of older vs. younger patients with a relative non-inferiority margin of 1.2.

The secondary endpoint “clinical response” and the baseline values of the 6 laboratory parameters cited previously were analyzed in the same way as the primary endpoint, using an ordinal logistic regression (testing the non-inferiority of older vs. younger) and linear regressions (testing the group effect: older vs. younger patients), respectively. Finally, the secondary endpoint OS (calculated from the start of the first PRRT intervention) was transformed from the standard time-scale to the reference-relative time-scale, which adjusts the survival time according to the residual life expectancy of a reference population [21,22], in this case, the Swiss population (using the appropriate life tables issued from the Swiss Federal Statistical Office). The group effect (older vs. younger patients) on the reference-relative OS was estimated by a Cox regression stratified by matching (the proportional hazards assumption of the Cox model was checked both graphically and numerically, by testing the correlation of the scaled Schoenfeld residuals with time). A non-inferiority test for older vs. younger patients as regards the reference-relative OS was performed by comparing the 95% confidence interval for the hazard ratio (HR) of older vs. younger patients with a value of 1.2 (i.e., the relative non-inferiority margin is 1.2).

## 3. Results

### 3.1. Patients and Baseline Characteristics

**Figure 1:**
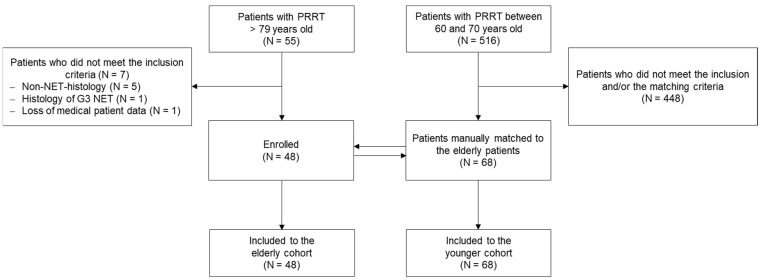
Study profile.

The study profile (flowchart) is given in Figure 1. Among the 55 patients >79 years who underwent at least one PRRT cycle at the University Hospital of Basel from January 2006 through December 2018, 48 fulfilled the inclusion criteria. All 48 patients who received at least 1 cycle were included in the safety analysis. Five hundred sixteen patients ranging from 60 to 70 years old underwent PRRT at the University Hospital of Basel during the same period from which 68 could be manually matched to the patients of the elderly cohort. Baseline demographics and clinical characteristics are summarised in Table 1. Except for age (y, mean ± SD: 81.7 ± 1.5 vs. 67.6 ± 1.7, EP vs. YP, respectively), both cohorts were well balanced. Midgut and pancreas were the most frequent primary tumour locations and all patients presented metastases, mainly in the liver, lymph nodes or bone. In the elderly cohort, 2.9 cycles per patient were administered on average, whereas the younger patients received 3.1 cycles. PRRT cycles with ^90^yttrium represented 23% (EP) and 30% (YP) (*p* = NS) of the treatments, respectively.

There was a tendency for EP to be more often pre-treated with PRRT (*p* < 0.001) and—as expected—to have more comorbidities (mainly cardiovascular (*p* = 0.025), renal (*p* = 0.018) and neurological (*p* = 0.003) disease) and a slightly lower ECOG status (*p* = NS) (Table 2). With regards to the treatment a trend was observed in which somatostatin analogues were less frequently administered in the EP (*p* = 0.002) (Table 2).

Table 3 gives the results of the estimated differences of the linear regressions for the baseline laboratory parameters: Compared with the YP, haemoglobin, total leukocytes, lymphocytes and in particular eGFR were—as expected—significantly lower in EP whereas thrombocytes and neutrophils were similar.

### 3.2. Safety

The results of the analyses of the subacute adverse events are given in Table 4. Twenty-two grade ≥ 3 or Δ ≥ 2 grades subacute hematotoxicities occurred in 10 elderly patients (13 PRRT cycles, i.e., in 10.3% of cycles) and 37 events occurred in 19 younger patients (23 PRRT cycles, 11.6% of cycles) (OR: 0.82 [0.28, 2.41], *p* = NS for non-inferiority of EP vs. YP). Six subacute grade ≥ 3 renal toxicities occurred in 6 EP (6 PRRT cycles, i.e., 4.9% of cycles) and 2 in 1 younger patient (2 PRRT cycles, 1.0% of cycles) (OR: 7.05 [0.02, 3213.47], *p* = NS for non-inferiority of EP vs. YP).

Table 5 shows the results for the long-term adverse events. Seven grade ≥ 3 or Δ ≥ 2 grades long-term hematotoxicities occurred in 7 elderly patients (20.0%) and 8 in 6 younger patients (14.0%) (OR: 1.54 [0.47, 5.10], *p* = NS for non-inferiority of EP vs. YP). Seven long-term grade ≥ 3 renal toxicities occurred in 7 elderly patients (20.0%) and 2 in 2 younger patients (4.5%) (OR: 5.25 [1.02, 27.14], *p* = NS for non-inferiority of EP vs. YP).

The results of the estimated differences did not change significantly if analytical statistics were calculated by multiple imputations instead of complete-case analyses.

### 3.3. Efficacy

**Figure 2:**
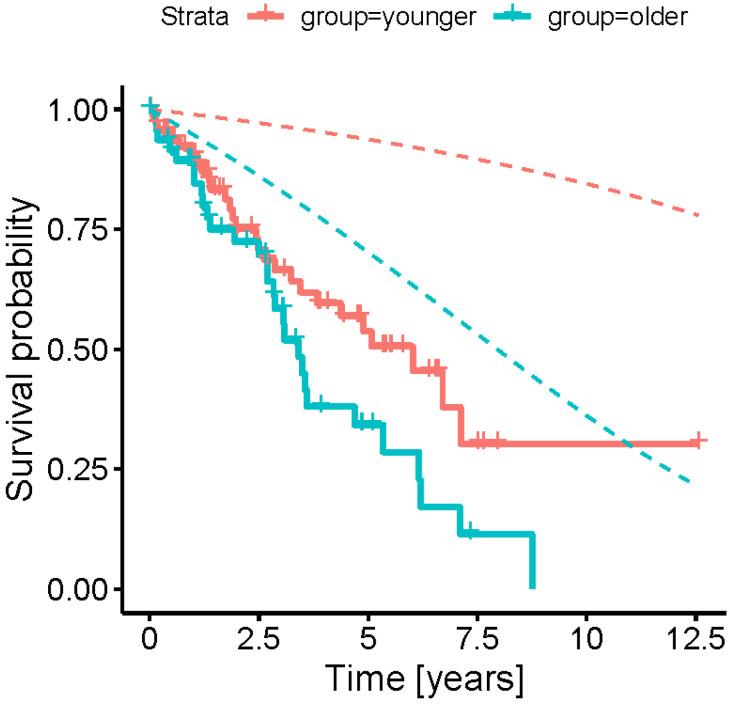
Kaplan–Meier plots comparing the OS in the two cohorts. The dashed lines represent the survival functions for the corresponding reference population. Both cohorts have a shorter OS than the reference population.

Overall, the survival of EP and YP was reduced compared to the healthy age- and gender-matched population, as anticipated (Figure 2). The median OS was 3.4 vs. 6.0 years (EP vs. YP, HR: 1.50 [0.75, 2.98], *p* = 0.094 for non-inferiority of younger vs. older patients). Therefore, YP tended to have an increase in survival benefit compared to the EP as expected. However, the median percentage of OS after adjusting for life-expectancy residual was 20.79 vs. 8.15% (EP vs. YP, HR: 0.41 [0.17, 0.96], *p* = 0.014 for non-inferiority of older vs. younger patients).

Fifty-three percent of our patients were non-Swiss, but most of them came from European countries. The reference for the age- and gender-adjusted OS was the Swiss population. However, the results did not change substantially if the patient’s individual country of origin was used as a reference population for calculation instead.

The results of the ordinal logistic regression for the estimated clinical response showed that the confidence intervals were too wide for showing non-inferiority of EP vs. YP (estimated OR: 1.16 [0.54, 2.52], *p* = NS). Differences between groups with respect to clinical benefit were not significant. But both cohorts had comparable high levels of clinical benefit according to their family physicians and NET specialists: moderate benefit: 10 EP (23.08%) vs. 17 YP (29.8%)/major benefit: 19 EP (45.2%) vs. 26 YP (45.6%). The return rates for physicians completing the questionnaire were 80% for the EP and 60% for the YP.

## 4. Discussion

The main results of this study can be summarised as follows: (1) Despite a reduced haematological functional reserve, a lower eGFR and more comorbidities with a trend for a reduced WHO/ECOG status at the beginning of therapy, PRRT did not result in increased toxicity in the EP. (2) Although OS was reduced in the EP and YP compared to the reference population, the reference-related time scale was not inferior in the EP compared to the YP. (3) The clinical benefit, as judged by the treating physicians, was high in both cohorts.

Epidemiological and demographic data suggest an increased incidence of NET and that the life span of the population in the western world increases [12,23]. It is, therefore, likely that the number of patients >79 years old with an advanced stage of NET will increase in the future and will therefore become progressively more relevant. For these potentially frail patients, it is critical to offer a therapy modality that is both well-tolerated and efficient. “Cold” somatostatin analogues are well-tolerated and have been shown to increase the time to progression in secreting [6] and progression-free survival in non-secreting NETs [3]. Our study shows that EP with functional activity were treated with somatostatin analogues for symptom control as frequently as the YP cohort; however, in the case of non-secreting NET, somatostatin analogues tended to be more frequently discontinued upon tumour progression prior to PRRT. In our opinion this is justified as, even though usually well-tolerated, somatostatin analogues may be associated with side effects such as diarrhoea, abdominal discomfort, nausea and flatulence and toxicity that should be avoided in possibly fragile patients.

Recently, the NETTER-1 trial has shown that PRRT is more effective in stabilising the disease compared to “cold” somatostatin analogues. However, the included patients had a mean age of 63 years—similar to our control group—and suffered from midgut NET [2]. Also, patient-reported tolerance to PRRT in a similar NET patients’ cohort is high [24]. However, it remained unclear whether similar results apply to elderly patients, in particular with regards to toxicity, which has not been investigated so far. To the best of our knowledge, our study is the first study looking specifically at the safety and efficacy of PRRT in elderly NET patients compared to a younger cohort with the typical age at which NET patients are diagnosed and treated. The results of our study suggest that subacute and long-term haematological and renal adverse events were similar in patients >79 years, compared to patients between 60 and 70 years old despite reduced functional haematological reserves, decreased eGFR at baseline, more comorbidities and a trend for reduced ECOG performance status. Consequently, PRRT can be considered as a safe therapeutical modality for patients >79 years with an advanced stage, well-differentiated NET.

The current data documents that the EP tended an increased frequency of re-treatment. Similarly, bone metastasis was more prevalent in the EP, consistent with a longer duration of disease. Despite these findings, toxicity, in particular bone marrow toxicity, was not increased in the EP, which is consistent with the fact that PRRT is safe also in an elderly patient population when careful patient selection is applied. However, due to the limited number of patients, we cannot fully exclude an increased risk of long term renal toxicity of PRRT in the EP compared to the YP cohort, especially when using ^90^Y-DOTATOC, reportedly more nephrotoxic than ^177^Lu-DOTATATE [18,25].

As expected, the OS was reduced in the younger and elderly cohort compared to the reference population. However, the age- and gender-adjusted curves point out that the OS of PRRT patients aged >79 years is not inferior compared to the patients aged 60 to 70 years old corroborating the hypothesis that PRRT is also a valid therapeutical modality for elderly patients with an advanced stage of well-differentiated NET.

There are inherent limitations based on the retrospective design of the study. Although the indications and contraindications for PRRT were consistent with the currently applicable recommendations [15,16,26], for both EP and YP groups, we cannot fully exclude a selection bias. Especially, we might overestimate the impact of PRRT on nephrotoxicity in the EP cohort as EP had a lower renal function at baseline. Conversely, we might underestimate it as ^90^Y-DOTATOC tend to be less used for the EP cohort as it is for the YP cohort reflecting on our attempt to mitigate the anticipated risk for long-term renal toxicity associated with ^90^Y-based PRRT regimen [18]. Due to the difficulties to assess response in NET in general—RECIST criteria are probably not ideal [27], the clinical benefits were judged—as a proxy—by the treating and referring physicians. It showed consistent and similar high benefits across both cohorts. If we assume that the judgement of clinical benefits includes the combination of efficacy and toxicity, the results of this study further substantiate the fact that PRRT may be an appropriate choice also for elderly patients.

Also, some patients underwent their first PRRT in 2006, and have died more than 10 years ago. It was, therefore, sometimes difficult to find valid follow-up data. Data completeness ranged from 78% to 94% and 53% to 87% for sub-acute and long-term toxicity outcome measures, respectively. However, the occurrence of missing data affected equally EP and YP which supports our findings. An actual assessment of the quality of life of the patients using questionnaires was not possible. Another limitation of the study was the relatively small patient population (total of 116 patients), which may limit the statistical analysis. However, the relatively low power of the study is partly explained by the low incidence of severe toxicity events, which supports our finding that PRRT in elderly patients is generally safe and similarly well tolerated compared to YP. Nevertheless, this study provides crucial information for the treatment of elderly patients with PRRT.

## 5. Conclusions

In summary, the current study indicates that PRRT in patients >79 years with advanced stage NET, who represent a significant and increasing part of our ageing population, is a valid therapeutic option with similar toxicity and non-inferior overall survival in comparison to PRRT in younger patients. However, an assessment of the benefits and risks should still be carefully balanced out and compared to alternative treatment modalities prior to a clinical decision in favour of PRRT.

## Figures and Tables

**Table 1:** Baseline patient demographics and clinical characteristics *.

Characteristic	Elderly Cohort (N = 48)	Younger Cohort (N = 68)
Age—years	81.7 ± 1.5	67.6 ± 1.7
Range—years	79–86	60–70
Sex—no. (%)		
Male	26 (54)	38 (56)
Female	22 (46)	30 (44)
Median time since diagnosis—y	4.3 ± 4.8	4.2 ± 6.0
Primary tumour site—no. (%)		
Midgut	21 (44)	28 (41)
Hindgut	3 (6)	3 (4)
Pancreas	11 (23)	19 (28)
Lungs	6 (13)	7 (10)
Unknown	7 (15)	11 (16)
Hormonal secretion—no. (%)		
Functional	11 (23)	17 (25)
Non-functional	37 (77)	51 (75)
Tumor grade—no. (%)		
Grade 1	29 (60)	37 (54)
Grade 2	19 (40)	31 (46)
Metastasis—no. (%)	48 (100)	68 (100)
Site of metastasis—no. (%)		
Liver	39 (81)	65 (96)
Lymph nodes	22 (46)	30 (44)
Bone	21 (44)	19 (28)
Peritoneum	11 (23)	6 (9)
Others	8 (17)	8 (12)
PRRT cycles—no. (per patient)	139 (2.9)	211 (3.1)
^177^lutetium—no. (%)	107 (77)	148 (70)
^90^yttrium—no. (%)	32 (23)	63 (30)
Retreatment—no. (%) **	14 (29)	17 (25)

Abbreviations: no., number; PRRT, peptide receptor radionuclide therapy; SD, standard deviation; y, years. * Plus-minus values are means ± SD. Percentages may not sum to 100 because of rounding. ** Retreatment = PRRT with 2–4 cycles after a first PRRT with 2–4 cycles.

**Table 2:** Baseline patient demographics and clinical characteristics *.

Characteristic	Elderly Cohort (N = 48)	Younger Cohort (N = 68)
Pretreatment—no. (%)		
PRRT	14 (29)	17 (25)
Surgery	21 (44)	28 (41)
Local ablative treatments	5 (10)	5 (7)
Chemotherapy	6 (13)	12 (18)
Radiotherapy	1 (2)	3 (4)
Targeted therapies	5 (10)	5 (7)
Other biotherapies	0 (0)	2 (3)
Somatostatin analogues	16 (33)	43 (63)
Comorbidities—no. (%)		
Cardiovascular	32 (67)	31 (46)
Renal insufficiency **	15 (31)	9 (13)
Diabetes	10 (21)	16 (24)
Neurological	6 (13)	0 (0)
Other tumors	9 (19)	10 (15)
WHO/ECOG Performance Status—no. (%)		
<2	38 (79)	60 (88)
=2	10 (21)	8 (12)

Abbreviations: no., number; PRRT, peptide receptor radionuclide therapy. * Percentages may not sum to 100 because of rounding. ** KDIGO CKD G2 (eGFR ≥ 60 and ≤ 89 mL/min/1.73 m^2^).

**Table 3:** Estimated mean differences of older vs. younger patients for the baseline values of laboratory parameters *, obtained by complete-case analyses, with confidence intervals and *p*-values for the difference of older vs. younger patients.

Laboratory Parameters	Elderly Cohort (N = 48 **)	Younger Cohort (N = 68 **)	Estimated Differences	95% CI	*p*
Haemoglobin [g/L]	126 ± 15	133 ± 12	−7.30	[−12.12, −2.47]	0.003
Thrombocytes [×10^9^/L]	249 ± 73	272 ± 85	−23.74	[−52.50, 5.01]	0.106
Leucocytes [×10^9^/L]	6.73 ± 1.61	7.60 ± 2.23	−0.87	[−1.61, −0.13]	0.022
Neutrophil granulocytes [×10^9^/L]	4.68 ± 1.53	5.32 ± 1.98	−0.64	[−1.32, 0.05]	0.070
Lymphocytes [×10^9^/L]	1.31 ± 0.54	1.49 ± 0.54	−0.19	[−0.37, −0.02]	0.032
eGFR (CKD-EPI) [mL/min/1.73 m^2^]	61 ± 16	78 ± 19	−17.70	[−24.43, −10.97]	<0.001

Abbreviations: CI, confidence interval; eGFR, estimated glomerular filtration rate; SD, standard deviation. *: Plus-minus values are means ± SD. **: The neutrophil granulocytes and lymphocytes counts were available in 45 and 62 patients in the elderly cohort and younger cohort, respectively.

**Table 4:** Estimated odds ratios of older vs. younger patients for subacute adverse events *, obtained by complete-case analyses, with confidence intervals for the non-inferiority of older vs. younger patients (with relative non-inferiority margin 1.2).

	CTCAE ≥ 3						CTCAE Δ ≥ 2					
	Elderly Cohort		Younger Cohort		OR	95% CI	Elderly Cohort		Younger Cohort		OR	95% CI
	Total No. of Events	% per PRRT Cycle	Total No. of Events	% per PRRT Cycle			Total No. of Events	% per PRRT Cycle	Total No. of Events	% per PRRT Cycle		
Anaemia	1	0.8	1	0.5	1.58	[0.10, 25.42]	1	0.8	4	2.0	0.39	[0.04, 3.51]
Thrombocytopenia	0	0.0	5	2.5	0.00	[0.00, Inf]	2	1.6	6	3.0	0.70	[0.01, 90.86]
Leukopenia	1	0.8	4	2.0	0.45	[0.00, 850.63]	11	8.7	17	8.6	1.01	[0.32, 3.23]
Neutropenia	1	0.9	2	1.1	0.82	[0.00, 127,175.27]	8	7.3	9	4.9	1.43	[0.05, 39.59]
Lymphopenia	27	25.0	36	19.9	1.46	[0.59, 3.64]	32	29.6	52	28.7	1.05	[0.60, 1.84]
Kidney disease	6	4.9	2	1.0	7.05	[0.02, 3213.47]						

Abbreviations: Δ, delta (increase in toxicity grade compared to baseline); CI, confidence interval; CTCAE, common terminology criteria for adverse events; no., number; OR, odds ratio; PRRT, peptide receptor radionuclide therapy. * Adverse events were defined according to the U.S. NCI Common Terminology Criteria for Adverse Events (CTCAE), version 5.0. Subacute = within 12 weeks after a cycle.

**Table 5:** Estimated odds ratios of older vs. younger patients for long-term adverse events *, obtained by complete-case analyses, with confidence intervals for the non-inferiority of older vs. younger patients (with relative non-inferiority margin 1.2).

	CTCAE ≥ 3						CTCAE Δ ≥ 2					
	Elderly Cohort		Younger Cohort		OR	95% CI	Elderly Cohort		Younger Cohort		OR	95% CI
	Total No. of Events	% per Patient	Total No. of Events	% per Patient			Total No. of Events	% per Patient	Total No. of Events	% per Patient		
Anaemia	0	0.0	0	0.0	1.00	[0.00, Inf]	5	14.3	5	11.6	1.27	[0.34, 4.78]
Thrombocytopenia	2	5.7	0	0.0	Inf	[0.00, Inf]	2	5.7	1	2.3	2.55	[0.22, 29.30]
Leukopenia	0	0.0	1	2.3	0.00	[0.00, Inf]	0	0.0	1	2.3	0.00	[0.00, Inf]
Neutropenia	0	0.0	1	2.5	0.00	[0.00, Inf]	0	0.0	1	2.5	0.00	[0.00, Inf]
Lymphopenia	6	18.2	3	7.5	2.74	[0.63, 11.94]	11	33.3	9	22.5	1.65	[0.58, 4.73]
Kidney disease	7	20.0	2	4.5	5.25	[1.02, 27.14]						

Abbreviations: Δ, delta (increase in toxicity grade compared to baseline); CI, confidence interval; CTCAE, common terminology criteria for adverse events; no., number; OR, odds ratio; PRRT, peptide receptor radionuclide therapy. * Adverse events were defined according to the U.S. NCI Common Terminology Criteria for Adverse Events (CTCAE), version 5.0. Long-term ≥ 1 year after PRRT.

## Data Availability

The data presented in this study are available on request from the corresponding author.

## Supplementary Materials

The following are available online at https://www.mdpi.com/article/10.3390/cancers13246290/s1, Figure S1: Standardised questionnaire for the treating physicians used for the clinical response assessment.
