# Supplementary material for: Safety and Efficacy of Peptide-Receptor Radionuclide Therapy in Elderly Neuroendocrine Tumor Patients
Source: Cancers (Basel). 2021 Dec 15;13(24):6290. doi: 10.3390/cancers13246290 (PMC8699207; doi:10.3390/cancers13246290)
Supplement: Supplementary file 1 [file cancers-13-06290-s001.zip › cancers-1459993-supplementary.pdf]

Universitätsspital Basel

Radiologie und Nuklearmedizin
Nuklearmedizin

Patient: **Last name, First name, Date of birth**

1. Is the patient still alive?

☐ Yes → Date of the last medical visit: ____________

☐ No → Date of death: ____________

Cause of death: ____________

2. Did the patient clinically benefit from the DOTATOC therapy?

☐	☐	☐	☐
no benefit	minor benefit	moderate benefit	major benefit

3. Did the patient receive any somatostatin analogues after completion of DOTATOC therapy?

☐ Yes ☐ No

Thank you for your efforts and collaboration.

Supplementary Figure 1. Standardized questionnaire for the treating physicians used for the clinical response assessment.
